# Iodide of Potassium in Tertiary Syphilis

**Published:** 1910-11

**Authors:** S. O. Young

**Affiliations:** Galveston, Texas


For Texas Medical Journal.

### Iodide of Potassium in Tertiary Syphilis.

BY S. O. YOUNG, M. D., GALVESTON, TEXAS.

I feel that it is presumptuous for me to express an opinion on any medical subject, and yet I can not resist the temptation to do so. The fact that I retired from the practice of medicine about thirty-five years ago should debar me, and yet when I review the history of medicine itself during those years, I do not feel so inclined to take a back seat.

I am much encouraged by realizing that, so far from being an exact science, medicine has undergone, and is still undergoing, wonderful changes. It is difficult for one who has floated along, placidly, with the current, to appreciate just how radical have been those changes; only one who has been resting quietly on the bank of the stream can understand them.

A few years ago I attended a meeting of the State Medical Association. I heard reports and listened to discussions and was greatly interested, of course; but barring those relating to surgery, with the literature of which I had kept more or less in touch, I have to confess that I recognized scarcely an old friend among the remedies, nor did I understand the whys and wherefores for the diagnoses. In my perplexity I sought an old friend who had been on "the firing line" all the time. He comforted me greatly by saying: "The papers you have heard read and the discussions you have listened to are 90 per cent 'dress parade.' It sounds big to speak of 'bacilli,' 'cocci,' 'anti this and anti that,' but the same old remedies you and I used years ago still hold good. In my experience I find that castor oil, calomel, quinine and hot mustard footbaths destroy just as many of the disease bugs as are destroyed by the new remedies so fashionable now."

From this discursive beginning you may be wondering what I am aiming at. The truth is, I am making my explanation and excuse in advance for aiming at anything at all through your columns, for I really feel that I have no right to express an opinion on any medical subject, and yet I am tempted all the time to do so. The present temptation comes from having read, in the October

TEXAS MEDICAL JOURNAL, that excellent article, "Syphilis and Its Treatment," by Dr. M. H. Simons, U. S. N., for it opened for me a new train of thought, based as it is on modern research and experience.

I was much instructed and greatly benefited by it, and yet after all I could not get away from impressions made on me when I, as resident student, had charge, from time to time, of the syphilitic wards of the Charity Hospital in New Orleans. At that time I was impressed by the fact that so many cases of syphilis coming into the hospital, after a course of treatment on the outside, showed almost always identical symptoms.

It was considered the proper thing at that time to cauterize the chancre and to at once place the patient on constitutional treatment. This "constitutional" treatment was looked on as a precautionary measure, and consisted of mercury, iodide of potassium and some bitter tonic. After noting the marked similarity of the symptoms in all the cases that came to the hospital I became convinced, and am convinced yet, that a very large per cent of so-called secondary syphilitic cases are nothing more than the effect of unwise and misdirected zeal on the part of the physician. A mixture of bichloride of mercury and iodide of potassium, if consistently poured into the strongest and healthiest man, will soon convert him into as fine a specimen of a secondary syphilitic case as one cares to see. I never had the heart to make the experiment, but I have seen several cases where there was no excuse for the treatment, and the result was always in accordance with my theory. I recall one case in particular; a young man who had five or six soft chancres. His physician insisted on "being on the safe side" and placed him on constitutional treatment. The result was a typical case of secondary syphilis.

Personally, I always refused to give more than local treatment in such cases, and never had bad results to follow. Dr. Simons speaks of specifics. Of course I know nothing of those he names, but from my personal experience I do know that iodide of potassium in heroic doses comes as nearly as possible being a specific in the third stage of the disease. I will give the history of one case to show the marvelous effect of such treatment.

I was treating a typhoid patient in an Irish boarding house when the proprietor asked me to see a poor fellow who, he said, was crazy and in a bad fix generally. I found him to be all the proprietor said, except being crazy. He had nodes on each tibia and two on his skull, one soft and pulsating. He had lost his hair and eyebrows and was a most repulsive sight. I saw at a glance that his condition was desperate and that desperate treatment was justified. I wrote a prescription ordering one drachm doses of iodide of potassium, taking the precaution to enclose a note to the druggist telling him that I wanted the prescription put up as I had written it. The treatment was begun at once, and its effect was marvelous, for exactly three weeks from the day the first dose was given the man, who was a section hand on a railroad, returned to work.

I treated other cases of the same character after that and always with success.

I have never met a syphilitic patient who had not undergone treatment either from a physician or who had taken some nostrum largely composed of iodide of potassium; therefore I attribute the specific action of the iodide in such cases as that mentioned above to the fact that the patient has been taking the drug for so long that it has lost its power either for good or evil and has become absolutely useless.

We know how useless it is to give a morphine fiend an ordinary dose of the drug. The same is true of all drugs after long use, therefore when one wishes to get beneficial effect from iodide of potassium, which has been taken by a patient for a long time, and which is so plainly indicated in the third stage of syphilis, one must greatly increase the dose.

---

For Texas Medical Journal.

